# Pancreaticoduodenectomy combined with splenectomy for a patient with pancreatic cancer and pancytopenia due to liver cirrhosis: Case report

**DOI:** 10.1016/j.ijscr.2021.105715

**Published:** 2021-03-04

**Authors:** Hideharu Tanaka, Hisashi Imai, Toshiya Higashi, Katsutoshi Murase, Nobuhisa Matsuhashi, Kazuhiro Yoshida

**Affiliations:** a Department of Surgical Oncology, Gifu University Graduate School of Medicine, 1-1 Yanagido, Gifu, Gifu, 501-1194, Japan; b Department of General and Cardiothoracic Surgery, Gifu University Graduate School of Medicine, 1-1 Yanagido, Gifu, Gifu, 501-1194, Japan

**Keywords:** Case report, Pancreaticoduodenectomy, Pancreatic cancer, Liver cirrhosis

## Abstract

**Introduction and importance:**

The incidence of patients with liver cirrhosis (LC) is increasing. Patients with LC are known to have a greater risk of postoperative morbidity and mortality than patients without LC. A treatment option such as pancreaticoduodenectomy (PD) has not been validated to be safe for these patients, especially those with pancytopenia due to portal hypertension (PH). Providing an effective treatment option for these patients is essential.

**Case presentation:**

Herein, we describe a patient with pancreatic cancer with pancytopenia due to LC that was successfully treated with PD combined with splenectomy. The patient was a 70-year-old woman who was referred to our hospital for evaluation of a mass in the pancreatic head after she developed obstructive jaundice. She was diagnosed with T2N0M0, Stage IB pancreatic cancer and pancytopenia due to PH associated with LC. She received 2 cycles of adjuvant gemcitabine/S-1 chemotherapy and underwent radical subtotal stomach-preserving pancreaticoduodenectomy with splenectomy to improve her pancytopenia. Histopathological examination of the resected specimen revealed an R0 resection showing an Evans grade IIa histological response. Her pancytopenia improved rapidly after surgery.

**Clinical discussion:**

Strict indications for PD, haemostatic control of intraoperative bleeding, and optimal perioperative management were important for preventing hepatic decompensation in this patient. Splenectomy is effective for thrombocytopenia due to LC; however, attention to postoperative complications such as overwhelming post-splenectomy infection and portal vein thrombosis is required.

**Conclusion:**

For patients with pancreatic cancer with pancytopenia due to LC, PD combined with splenectomy plus optimal perioperative management is effective.

## Highlights

• Patients with LC are known to have a greater risk of postoperative morbidity and mortality than patients without LC.
• The outcomes of surgery in patients with LC have been reported to vary, based not only on the degree of damage to the liver but also the invasiveness of the surgery.
• For patients with PC with pancytopenia due to LC, PD combined with splenectomy is effective.

## 1 Introduction

Patients with liver cirrhosis (LC) have been recognized to have a greater risk of postoperative morbidity and mortality than patients without LC [1,2]. The safety of pancreaticoduodenectomy (PD) for patients with chronic hepatic dysfunction due to LC has not been validated. The risk of pancreaticoduodenectomy (PD) for patients increases with the progression of LC, because of the development of pancytopenia due to portal hypertension (PH) and secondary hypersplenism.

Perioperative chemotherapy has played an increasingly important role in the treatment of patients with pancreatic cancer (PC). Therefore, it is important to provide appropriate treatment options, including chemotherapy, for a patient with PC and pancytopenia due to LC. Herein we describe a patient with PC with pancytopenia due to LC who was successfully treated by PD combined with splenectomy, which enabled the safe postoperative administration of chemotherapy. We report this case in accordance with the 2020 SCARE criteria [3].

## 2 Case report

A 70-year-old woman with obstructive jaundice underwent percutaneous transhepatic biliary drainage instead of endoscopic biliary drainage because of her duodenal stenosis. She was then referred to our hospital for evaluation of a pancreatic head lesion that had been identified on an abdominal computed tomography (CT) scan performed when her jaundice had been discovered. She had a past history of endovascular aneurysmal repair (EVAR) for an abdominal aortic aneurysm one year previously and an appendectomy followed by surgery for an ileus when she was 10 years of age. Her family history was negative for pancreatic cancer and genetic disorders. She was 151 cm tall and weighed 60 kg. Her body-mass index was 26.3. The results of her physical examination were unremarkable.

**Fig. 1:**
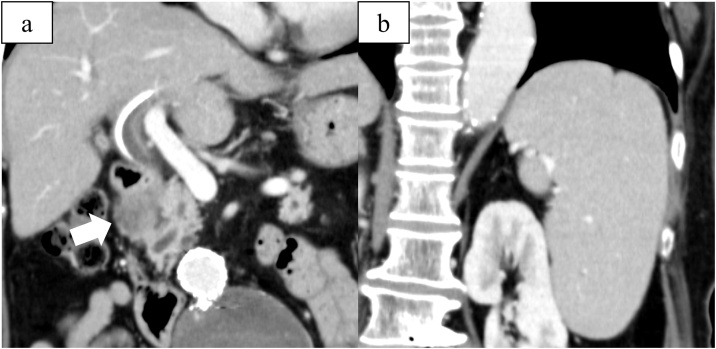
Pretreatment coronal enhanced computed tomography images. a) Hypovascular 25-mm tumour (white arrow) in the pancreatic groove invading the duodenum and lower bile duct. b) Splenomegaly with a maximum splenic diameter of 15 cm, which suggests portal hypertension.

Laboratory analysis revealed pancytopenia (white blood cell count 2710/μL, red blood cell count 366 × 10^4^/μL, haemoglobin 10.9 g/dL, platelet count 89 × 10^3^/μL) and CA19−9 3909 U/mL). She was negative for hepatitis B surface antigen and anti-hepatitis C virus antibody. Her Child-Pugh score and Model for End-stage Liver Disease (MELD) score were Grade 5A and 9, respectively [[4], [5], [6]]. Enhanced multidetector-row computed tomography (CT) revealed a 25-mm hypovascular tumour in the pancreatic groove, which had invaded the duodenum and lower bile duct (Fig. 1a). No other metastases involving distant organs were seen. Dullness of the hepatic margins was observed, and the spleen appeared enlarged, with a maximum diameter of 15 cm, suggesting portal hypertension (PH) (Fig. 1b). Endoscopic ultrasound-guided fine needle aspiration was performed. The cytopathological findings revealed pancreatic adenocarcinoma. Based on the 8th edition of the UICC criteria, the findings were diagnosed to be T2N0M0, Stage IB pancreatic carcinoma.

In accordance with the findings of the phase 2/3 Prep-02/JSAP05 trial [7], the patient underwent neoadjuvant gemcitabine/S-1 (GS) chemotherapy (gemcitabine [800 mg/m^2^] on days 1, 8 and S-1 [100 mg/m^2^] on days 1–14 of a 21-day cycle) repeated every 3 weeks for 2 cycles. Because of the patient’s pretreatment pancytopenia (neutrophil count 2250/μL), gemcitabine and S-1 were reduced by 200 mg/m^2^/day to 800 mg/m^2^/day, and 20 mg/m^2^/day to 100 mg/m^2^/day, respectively. Even after the dose reduction, neutropenia (neutrophil count 1340/μL) was observed during 1 cycle of GS, and chemotherapy was withheld until the neutropenia was resolved. Although the patient’s serum CA 19−9 level decreased after 2 cycles of GS chemotherapy, it remained elevated (CA 19−9 1064 U/mL). After 2 cycles, the primary tumour had slightly decreased to a diameter of 24 mm. Since the level of her CA19−9 tumour marker remained elevated, her risk of postoperative recurrence after PD was high. However, if the tumour recurred, the administration of intensive chemotherapy such as FOLFIRINOX (leucovorin and fluorouracil plus irinotecan and oxaliplatin) and gemcitabine/nab-paclitaxel regimens would be difficult because of the patient’s pancytopenia. Therefore, we elected to perform a splenectomy in addition to the PD to improve her pancytopenia.

Splenectomy for splenomegaly with PH occasionally results in loss of a large amount of blood, which requires a large transfusion. The transfusion leads to oedema of the intestinal wall, which can increase the difficulty of performing a PD. Therefore, we decided to perform the PD followed by splenectomy.

**Fig. 2:**
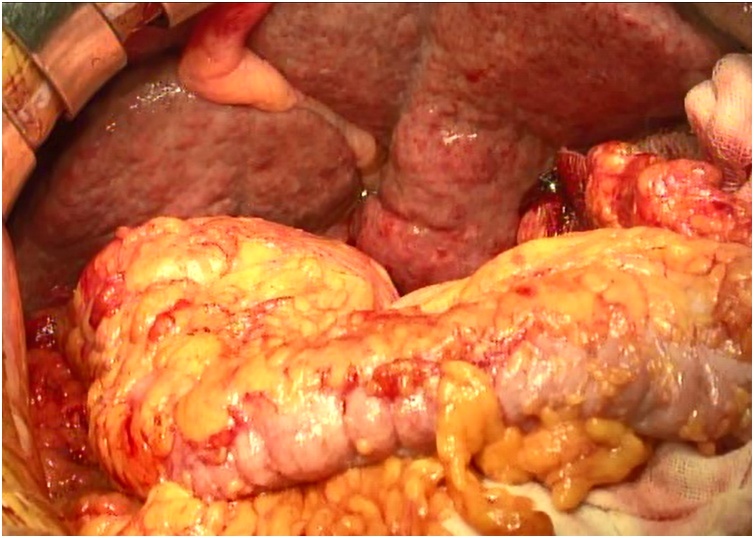
Intraoperative image. Intraoperative findings show the dullness of the liver’s edge and its granular surface.

The intraoperative findings revealed a liver with a granular surface and dullness of the liver’s edge (Fig. 2). We performed extensive detachment of the adhesions due to the patient’s previous appendectomy and subsequent surgery for an ileus, and found that detaching the hepatoduodenal ligament was difficult because of her repeated preoperative episodes of cholangitis. Since the collateral hepatic circulation that had developed bled easily, haemostasis was performed safely by ligation. After the PD, we began the splenectomy. We first detached and ligated the splenic artery (SA) at the upper edge of the pancreatic body to shrink the spleen and reduce the amount of blood loss. The splenic vein (SV) was also ligated, but the splenic hilum bled heavily because of tension on the SV. Haemostasis was performed by ligation while elevating the splenic hilum from the dorsal side. Finally, we performed a radical subtotal stomach-preserving PD with splenectomy, and lymph node dissections of groups 1 and 2 that included the hepatoduodenal ligament caudal to the hilar plate. The operative time was 12 h and 7 min, and the volume of blood loss was 1990 mL.

**Fig. 3:**
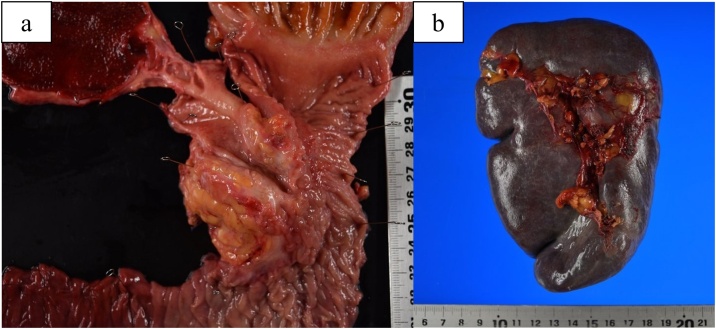
Macroscopic views of the resected specimens. a) Macroscopic image of the resected specimen shows a mass in the pancreatic head, which has invaded the duodenum and bile duct. b) The resected spleen is enlarged.

**Fig. 4:**
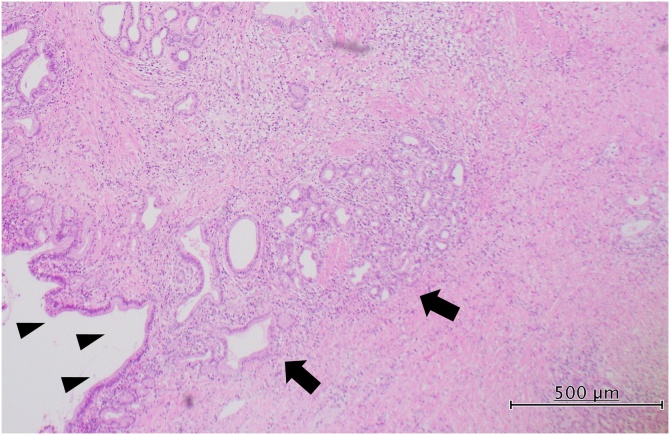
Photomicrograph (haematoxylin-and-eosin stain). Malignant cells (arrows) have invaded the bile ducts (arrowheads).

The excised specimens showed a solid tumour in the pancreatic head which had invaded the duodenum and bile duct (Fig. 3a), and splenomegaly (Fig. 3b). The histopathological diagnosis was invasive ductal carcinoma with severe venous invasion and moderate nerve infiltration, ypT2N0M0, ypStage IB pancreatic cancer, as based on the 8th Edition of the UICC criteria and grade IIa as based on the Evans classification [8] (Fig. 4).

**Fig. 5:**
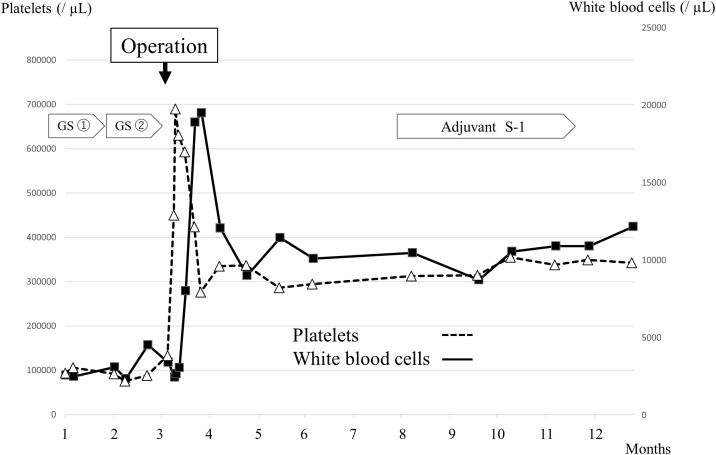
Graph of platelet and white blood cell counts over the patient’s hospital course. GS: Neoadjuvant gemcitabine/S-1 chemotherapy Adjuvant S-1: Adjuvant S-1 chemotherapy

The patient’s pancytopenia improved rapidly after surgery. The platelet count increased rapidly after splenectomy, peaking at 681,000/μL on postoperative day (POD) 16, and then decreasing to the normal range (Fig. 5). However, the patient developed atelectasis and splenic vein thrombosis, which were considered Grade II complications in accordance with the Clavien-Dindo classification. The patient received high-flow nasal oxygen therapy and noninvasive positive-pressure ventilation, including bilevel positive airway pressure, and was prescribed an anticoagulant agent for the thrombosis. The patient was moved out of the intensive care unit on POD 5, and discharged on POD 30. After having received adjuvant chemotherapy with S-1 for 2 months due to the anorexia, the patient underwent blood tests and CT scans every 3 months. Nine months after her PD, a liver metastasis was detected, for which she received chemotherapy. She has remained alive for 13 months after her PD.

## 3 Discussion

The outcomes of surgery in patients with LC have been reported to vary, based not only on the degree of damage to the liver but also the invasiveness of the surgery [9]. The reported mortality of major abdominal surgery for patients with LC is 35% [10]. The Child-Pugh classification and the MELD score have been useful for estimating the risk of surgical mortality. According to the Child-Pugh classification, the mortality rates for major abdominal surgery have been 10%, 30%, and 76%–82% for Child-Pugh classifications A, B, and C, respectively [11]. Whereas, according to the MELD score, the 30-day mortalities after surgery were reported to be 6% for MELD < 8 and 50% for MELD > 20 [12]. However, until now, to our best knowledge, only a few studies have been published on the outcomes of PD in patients with LC. A retrospective multicentre study reported that PH and a serum aspartate aminotransferase (AST) of <50 IU/L were significant independent risk factors for hepatic decompensation, and an AST of < 50 IU/L and an AST-to-platelet-ratio index (APRI) ≥ 1.0 for a patient with a Child-Pugh B classification may be indications that PD can be performed [13]. At the first visit, our patient’s AST value, APRI value, and Child-Pugh score were 57 IU/L, 1.6, and 5A, respectively. After 2 cycles of GS, her preoperative AST and APRI values had decreased to 22 IU/L and 0.5, respectively. Therefore, our patient had appropriate indications for undergoing a PD. Her postoperative Child-Pugh score has remained 5–6 (Class A), with no increase in the APRI (Table 1). The increase in the postoperative MELD score was considered to be due to her anorexia associated with S1 adjuvant chemotherapy.

**Table 1:** Table of perioperative Child-Pugh and MELD scores and APRI score.

	Pre-treatment	After 2 cycles of GS	POM1	POM2	POM3	POM4	POM5	POM6
Child-Pugh score	5	5	5	5	5	6	6	5
MELD score	9	3	1	0	1	3	5	3
APRI	1.6	0.5	0.2	0.1	0.1	0.1	0.1	0.1

APRI: AST-to-platelet-ratio index.

GS: Neoadjuvant gemcitabine/S-1 chemotherapy.

POM: Postoperative month.

Pancytopenia has a wide range of aetiologies, including malignancies (leukaemia, malignant lymphoma, myelodysplastic syndrome, myeloma), autoimmune conditions (aplastic anaemia, systemic lupus erythematosus [SLE]), hypersplenism due to LC, drugs, infections, and nutritional deficiencies, so the evaluation of a patient with pancytopenia requires a comprehensive approach [14]. Regarding the patient’s thrombocytopenia, the causes of thrombocytopenia often overlap with the causes of pancytopenia [15]. Because our patient did not have lymphadenopathy, SLE, recent extensive thrombosis, blasts or atypical cells at peripheral blood tests, abnormal findings in her bone marrow, abnormal lymph nodes on CT or PET-CT, signs of infection, recent changes in oral medication, or a history of gastrectomy, we excluded malignancies, autoimmune conditions, drug reactions, infections, and nutritional deficiencies from the differential diagnosis. CT showed dullness of the hepatic margins, splenomegaly, and presence of a collateral circulation; we concluded that her pancytopenia, including her thrombocytopenia, was caused by hypersplenism due to LC. Therefore, we did not think that a bone-marrow biopsy was a priority, compared with preoperative chemotherapy for pancreatic cancer.

Thrombocytopenia (platelet count < 150,000/μL) [16] can occur with the progression of LC. The production of thrombopoietin and sequestration of platelets in the spleen are the main mechanisms involved in the development of thrombocytopenia [17]. The treatments for thrombocytopenia due to LC have included platelet transfusions, medications, embolization of the SA, and splenectomy [18]. With our patient’s elevated CA19−9 level, the risk of recurrence of her pancreatic cancer was considered high, and we elected to add treatment for improving her pancytopenia in order to enable the safe administration of chemotherapy after her recurrence. Platelet transfusion was not performed because of her splenomegaly and because of the possibility of platelet sequestration followed by destruction. We did not administer a thrombopoietin receptor agonist such as eltrombopag, which is effective for thrombocytopenia, because the patient required treatment not only for her thrombocytopenia but also for her pancytopenia. Whether a partial splenic artery embolization (PSAE) or splenectomy should have been performed has been controversial. The complications of PSAE include peritonitis, splenic abscess, and portal vein thrombosis. Additionally, PSAE is an invasive procedure, with both significant cost and uncertain long-term benefits. These limitations make PSAE an unsuitable option for many patients with advanced LC [17]. Furthermore, Miyake et al. reported that platelet counts after splenectomy were significantly increased over platelet counts after PSAE [19]. On the other hand, although splenectomy is an effective option for improving pancytopenia, it is also invasive and occasionally results in massive loss of blood. Additionally, the main complications after splenectomy are overwhelming post-splenectomy infections (OPSIs), approximately 80% of which are pneumococcal, and portal vein thrombosis. The reported incidence of portal vein thrombosis after splenectomy for splenomegaly has ranged from 9% to 29% [17].

For our patient, we debated about performing a splenectomy. Ligation or embolization of the SA was also considered, but we considered the effect of those procedures to be limited, because the blood supply of the spleen was delivered via collateral circulation, including the left gastric artery, the stomach wall, and the middle colic artery [20,21]. Therefore, we decided to perform a splenectomy, which seemed to provide the most reliable effect of increasing the blood cell count for the chemotherapy after recurrence of pancreatic cancer [19]. After confirmation that there were no distant metastases after neoadjuvant chemotherapy, we finally performed a radical PD combined with splenectomy following the approval by a multidisciplinary team a few days before the surgery. The patient also received postoperative pneumococcal vaccine to prevent OPSI before she underwent chemotherapy. However, the vaccine should have been administered before surgery in this high-risk patient.

With regard to preoperative chemotherapy, Karl et al. reported the usefulness and low toxicity of chemotherapy administered by intra-arterial infusion, including isolated abdominal perfusion [22]. However, our patient also had an abdominal aortic aneurysm with type 2 endoleak despite her previous EVAR, so catheterization was considered to be difficult. Additionally, her pretreatment CA19−9 level was very high (CA19−9 3909 U/mL), suggesting the possibility of micrometastases. Therefore, we elected to perform dose-reduced systemic chemotherapy for the early treatment and suppression of micrometastatic disease.

To our best knowledge, when limited to cases of PD synchronous with splenectomy for PC with pancytopenia due to LC, a total of 2 cases have been reported, including our case [23]. As in our patient, who lost a large volume of blood, Futagawa et al. reported a large blood loss (1700 mL). In patients with LC, bleeding occurs readily because of PH and decreased coagulability, and there is concern that hepatic function may deteriorate because of blood transfusions. Therefore, the control of intraoperative bleeding by reliable haemostasis is important. Furthermore, in patients with PH in whom collateral circulation develops, attention must be paid to the extent of lymph node dissection. Dissection of the hepatoduodenal ligament interrupts the collateral circulation, which can lead to refractory ascites and hepatic decompensation. Group 1 and 2 lymph node dissections were performed for our patient, who did not develop hepatic decompensation. However, careful planning that takes into consideration a patient’s overall condition and degree of liver damage is needed before surgery for these patients.

When PD is performed for a patient with LC, prevention of hepatic decompensation, which is directly is associated with mortality, is the most important consideration. Optimal perioperative management may decrease morbidity and mortality following surgery. Perioperative management for patients with LC includes nutritional management, control of refractory ascites and glucose levels, and correction of coagulopathy. These measures can help in preventing infection, hepatic encephalopathy and hepatic decompensation [24]. In our case, enteral nutrition therapy was started immediately after surgery for nutritional management and the prevention of postoperative infections [25]. In addition, since patients with LC who undergo PD, which is a highly invasive procedure, have increased risk of postoperative morbidity and mortality, careful monitoring and follow up are essential.

## 4 Conclusions

Strict indications for PD, control of intraoperative bleeding, and optimal perioperative management are important for preventing hepatic decompensation. Even for patients with PC and pancytopenia due to LC, PD combined with splenectomy is an effective treatment option.

## Declaration of Competing Interest

The authors declare that they have no competing interests.

## Funding

This study did not receive any specific grants from funding agencies in the public, commercial, and not-for-profit sectors.

## Ethical approval

This study was exempt from ethical approval at our institution.

## Consent

Written informed consent was obtained from the patient for publication of this case report and accompanying images. A copy of the written consent is available for review by the Editor-in-Chief of this journal on request.

## Author contribution

HT (certified by the Japanese Society of Gastroenterological Surgery) performed the surgery on this patient. IH, HT, KM, and TH managed the postoperative course of this patient. HT and HI wrote the manuscript. All authors have read and approved the final manuscript.

## Registration of research studies

Not Applicable.

## Guarantor

Hideharu Tanaka (Corresponding author and guarantor).

## Provenance and peer review

Not commissioned, externally peer-reviewed.

## Availability of data and materials

Not applicable.
